# The detrimental effects of intestinal injury mediated by inflammation are limited in cardiac arrest patients: A prospective cohort study

**DOI:** 10.1016/j.resplu.2024.100639

**Published:** 2024-04-17

**Authors:** Bjørn Hoftun Farbu, Stian Lydersen, Randi Marie Mohus, Thor Ueland, Tom Eirik Mollnes, Pål Klepstad, Halvor Langeland

**Affiliations:** a Department of Anaesthesiology and Intensive Care Medicine, St. Olav’s University Hospital Trondheim, Norway; b Institute of Circulation and Medical Imaging, Faculty of Medicine and Health Sciences, Norwegian University of Science and Technology (NTNU), Trondheim, Norway; c Norwegian Air Ambulance Foundation, Department of Research and Development, Oslo, Norway; d Regional Centre for Child and Youth Mental Health and Child Welfare, Department of Mental Health, Faculty of Medicine and Health Sciences, Norwegian University of Science and Technology (NTNU), Trondheim, Norway; e Thrombosis Research Center (TREC), Division of Internal Medicine, University hospital of North Norway, Tromsø, Norway; f Institute of Clinical Medicine, University of Oslo, Oslo, Norway; g Research Institute of Internal Medicine, Oslo University Hospital (Rikshospitalet), Oslo, Norway; h Department of Immunology, Oslo University Hospital and University of Oslo, Oslo, Norway; i Research Laboratory, Nordland Hospital, Bodø, Norway

**Keywords:** Cardiac arrest, Intestinal ischaemia, Intestinal fatty acid binding protein, Multiple organ dysfunction, Inflammation, Mediation

## Abstract

**Background:**

Ischaemic intestines could be a driver of critical illness through an inflammatory response. We have previously published reports on a biomarker for intestinal injury, plasma Intestinal Fatty Acid Binding Protein (IFABP), and inflammatory biomarkers after out-of-hospital cardiac arrest (OHCA). In this post-hoc study we explored the potential indirect effects of intestinal injury mediated through the inflammatory response on organ dysfunction and mortality.

**Methods:**

We measured IFABP and twenty-one inflammatory biomarkers in 50 patients at admission to intensive care unit after OHCA. First, we stratified patients on median IFABP and compared biomarkers between “low” and “high” IFABP. Second, by causal mediation analysis, we assessed effects of IFABP through the two most important inflammatory biomarkers, interleukin (IL)-6 and terminal complement complex (TCC), on day two circulatory variables, Sequential Organ Failure Assessment (SOFA)-score, and 30-day mortality.

**Results:**

Cytokines and complement activation were higher in the high IFABP group. In mediation analysis, patients on the 75th percentile of IFABP, compared to the 25th percentile, had 53% (95% CI, 33–74; *p* < 0.001) higher risk of dying, where 13 (95% CI, 3–23; *p* = 0.01) percentage points were mediated through an indirect effect of IL-6. Similarly, the indirect effect of IFABP through IL-6 on SOFA-score was significant, but smaller than potential other effects. Effects through IL-6 on circulatory variables, and all effects through TCC, were not statistically significant and/or small.

**Conclusion:**

Effects of intestinal injury mediated through inflammation on organ dysfunction and mortality were limited. Small, but significant, effects through IL-6 were noted.

**Trial registration**: ClinicalTrials.gov: NCT02648061.

## Introduction

After being initially successfully resuscitated from cardiac arrest, two-thirds of patients suffer from severe multiple organ dysfunction. Mortality increases with severity of extra-cerebral organ dysfunction and is around 90% in the most severe cases.^1^, ^2^, ^3^ While two-thirds die of neurological injury overall, cardiovascular dysfunction is the most common cause of death the first three days.^4^ The mechanisms leading from whole-body ischaemia to multiple organ dysfunction and death have not been determined, but the immune system is suggested to play a central role.^5^ Moreover, intestinal ischaemia might be a “driver” of the subsequent inflammatory response and multiple organ dysfunction.^6^, ^7^ Indeed, when and how intestinal injury leads to multiple organ dysfunction are highlighted as research questions by the European Society of Intensive Care Medicine.^8^

Both plasma Intestinal Fatty Acid Binding Protein (IFABP), a biomarker for small bowel injury, and inflammatory biomarkers, including cytokines and complement activation products, are elevated after cardiac arrest.^5^, ^9^, ^10^, ^11^ The origin of inflammatory biomarker release is not established, but some studies suggest that ischaemic intestines might initiate the inflammatory cascade.^12^, ^13^, ^14^, ^15^ However, there are few reports investigating the associations between intestinal injury and systemic inflammatory biomarkers in general.^15^, ^16^, ^17^, ^18^, ^19^ Previously, we have reported that both IFABP and some of the analysed inflammatory biomarkers, including interleukin (IL)-6 and the terminal complement complex (TCC), were associated with multiple organ dysfunction and mortality after out-of-hospital cardiac arrest (OHCA).^20^, ^21^

Causal mediation analysis is an emerging statistical method which contributes to new insights into pathophysiologic processes.^22^ The method combines conventional regression models and enables a decomposition of the total effect of an exposure on outcome into an indirect effect through a mediator and a direct effect which is not attributed to the mediator. To draw firm causal conclusions, rigorous assumptions are required.^23^, ^24^

The aim of this study was to explore, by causal mediation analysis, the potential indirect effects of intestinal injury mediated through the inflammatory response on organ dysfunction and mortality after out-of-hospital cardiac arrest (OHCA).

## Methods

### Study design and setting

This is a post hoc analysis of a prospective cohort included at St. Olav’s University Hospital, Norway. The inclusion period was January 2016 to November 2017. In addition to the inflammatory biomarkers (including IL-6 and TCC) and intestinal injury (IFABP), reports on circulatory characteristics in this cohort have been published previously^20^, ^21^, ^25^, ^26^. The AGReMA Statement for mediation analysis reporting published in JAMA, guided this study report.^27^

### Participants

Inclusion criteria were successfully resuscitated patients admitted to the Intensive Care Unit (ICU) after OHCA. Exclusion criteria were age < 18 years, pregnancy, sepsis or anaphylaxis as assumed causes of arrest, transferal from other hospitals, decision upon arrival to limit life-sustaining therapy, or the following before admission to the ICU: application of extracorporeal membranous oxygenation (ECMO), or a ventricular assist device (VAD), or cardiothoracic surgery. Patients were followed for five days or until they were transferred to ward or died, ECMO or VAD was applied, cardio-thoracic surgery was performed, or withdrawing of life-prolonging therapies was decided. Day “zero” lasted from admission until next morning at 06:00, and thus had variable length.

### Initial management

Targeted temperature management (TTM) at 36 °C for 24 hours was the standard treatment of comatose patients. Patients presenting with clinical signs of hypoperfusion and/or hypotension were given fluids, vasopressors and/or inotropic drugs. If indicated, percutaneous coronary intervention was done. Comprehensive information regarding the clinical care has been reported earlier.^28^

### Data sources and measurement

We gathered data from the pre-hospital report in accordance with the Utstein cardiac arrest template, and information on assessment and treatment from the hospital record.^29^ We scored Sequential Organ Failure Assessment (SOFA) daily.^30^ Lactate was obtained from the first arterial blood gas sample after admission.

In all comatose patients without contraindications, we inserted a pulmonary artery catheter (Swan-Ganz CCOmbo, Edwards Lifesciences, Irvine, CA) for continuous haemodynamic measurements. We obtained all circulatory variables and medications from the electronic critical care information system (Picis CareSuite, Optum Inc. Peabody, MA). The circulatory variables were mean arterial pressure (MAP), cardiac output (CO), systemic vascular resistance (SVR), dose of noradrenaline (norepinephrine), and amount of fluid infusion. MAP, CO, SVR and dose of noradrenaline presented are the mean values over the period beginning 30 minutes before each blood sampling and ending 30 minutes after. Amount of fluid given is the mean for the previous 24 hours. We obtained 30-day mortality from the medical records and the Norwegian death registry.

### Biomarkers

#### Blood sampling and plasma preparation

Blood samples were drawn at inclusion and every morning, gently mixed, placed vertical in ambient temperature for 30 minutes and then centrifuged at 2200*g* for 10 minutes. Within 1 hour from sampling, EDTA-plasma was frozen to −80 °C.

#### Intestinal Fatty Acid Binding Protein (IFABP), endothelium and platelet markers

Plasma levels of IFABP, syndecan-1, vascular endothelial (VE)-cadherin and von Willebrand factor (vWF) were measured in duplicate by enzyme immunoassays (EIA) using commercially available antibodies (Cat# DY3078, R&D Systems, Minneapolis, MN) in a 384-format using a combination of a CyBi-SELMA pipetting robot (Analytik Jena, Germany) and an automatic dispenser/washer (BioTek, Winooski, VN). Absorption was registered at 450 nm with wavelength correction set to 540 nm applying an ELISA plate reader (BioTek).

#### Complement activation and cytokines

Initial C3 activation product (C3bc) and TCC were measured by ELISA, applying the Complement International Standard #2.^31^ Plasma levels of the following cytokines were analysed with Bio-Plex ProTM Human Cytokine 27-plex Assay (Bio-Rad, Hercules, CA): IL-1 receptor antagonist (IL-1ra), IL-6, IL-8, regulated on activation normal T-cell expressed and secreted (RANTES).

### Statistical methods

Descriptive statistics are presented as mean (standard deviation [SD]), median (interquartile range [IQR]), or proportion (%). Of twenty-one biomarkers in the previous report, biomarkers consistently associated with outcome were included in the present (TCC, C3bc, IL-1ra, IL-8, IL-6, RANTES, Syndecan-1, VE-cadherin and vWF), and only the two variables associated with mortality in the multivariable analysis (IL-6 and TCC) were included in the mediation analyses.^20^ We used admission values of all biomarkers. IL-6 had one outlier which was changed to “missing” and omitted in the analyses.

To assess the associations between inflammatory biomarkers and intestinal injury, we divided the cohort in “low” and “high” by the median IFABP. Biomarkers were compared between the two groups using the Student t-test or the Wilcoxon rank-sum test. We displayed survival and the values of biomarkers first five days graphically.

#### Mediation analyses

**Fig. 1:**
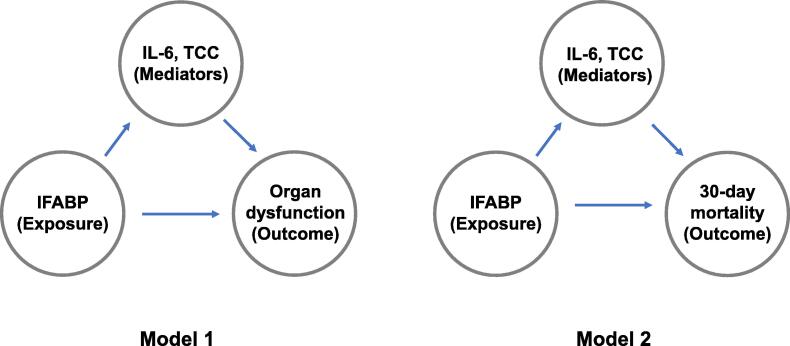
The two mediation models. IFABP is the exposure and inflammatory biomarkers are mediators in both models. Organ dysfunction is the outcome in Model 1 and 30-day mortality is the outcome in Model 2. Potential confounding factors such as whole-body ischaemia could affect all variables. IFABP: Intestinal Fatty Acid Binding Protein. IL: Interleukin; TCC: terminal complement complex.

To assess the potential effects of IFABP through inflammatory biomarkers on organ dysfunction and death, we applied causal mediation analysis.^22^, ^24^ This approach is based on the potential outcomes framework and is implemented in Stata (StataCorp.2023. *Stata 18 Causal mediation analysis Manual*. Stata Press, College Station, TX). The potential *total effect* of an exposure on outcome is the sum of the *natural indirect effect* through a mediator and *the natural direct effect*, in the text simply named the “indirect” and “direct” effect. To make valid causal conclusions, these assumptions also must be fulfilled: There must be no unmeasured confounding in the exposure-outcome, the exposure-mediator, or the mediator-outcome relationships. In addition, there must be no confounders in the mediator-outcome relationship caused by the exposure. The mediation analyses, with assumed causes and effects, are presented in Fig. 1.

We applied two mediation models using organ dysfunction (Model 1) and 30-day mortality (Model 2) as the outcomes. The indirect effect was our main interest. In both models IFABP was set as the exposure, and IL-6 and TCC were log_2_ transformed and entered one by one as mediators. Organ dysfunction at start of day two was evaluated by six outcome variables separately (SOFA-score, SVR, MAP, CO, dose of noradrenaline and amount of fluid given). Linear regression was applied for both the mediator and outcome functions in Model 1. In Model 2, we applied a probit-function for the dichotomous outcome. The effects (total, direct and indirect) were expressed as the regression coefficients (Model 1) and risk difference (RD) (Model 2) between the 75th percentile of IFABP, compared to the 25th percentile, with 95% confidence intervals (CI).

In Model 2, with IL-6 as the mediator, we included covariates that were plausible confounders (i.e. could affect both the exposure–mediator association and the mediator–outcome association).^24^ These covariates were initial non-shockable rhythm, time to return of spontaneous circulation (ROSC) and initial lactate at hospital admission. We added the covariates one at a time because of the low sample size. The Stata commands are displayed in Supplementary Table 1.

#### Sensitivity analysis

We performed several sensitivity analyses. First, we exchanged admission levels for day one levels of IL-6. Second, we applied two logistic regression models with IFABP, IL-6 and TCC as independent variables. In the first regression model, 30-day mortality was evaluated with confounding factors added one at a time, and in the second by excluding patients who were considered to die of irreversible brain injury.^21^

This was a descriptive study, and no sample size was calculated.^28^ All tests were two-sided and statistical significance was defined as *p* < 0.05. We did not adjust for multiple testing. Data were extracted with the software Matlab (Mathworks Inc., Natick, MA), and the statistical analyses were performed with Stata 18.0 (StataCorp LCC, Collage Station, TX).

### Ethics

The Regional Committee for Medical and Health Research Ethics, Central Norway Health Region (REK Midt, No. 2015/1807) approved the study. Participants or their proxies provided written consent. The study is registered in ClinicalTrials.gov (Identifier: NCT02648061).

## Results

### Participants

**Fig. 2:**
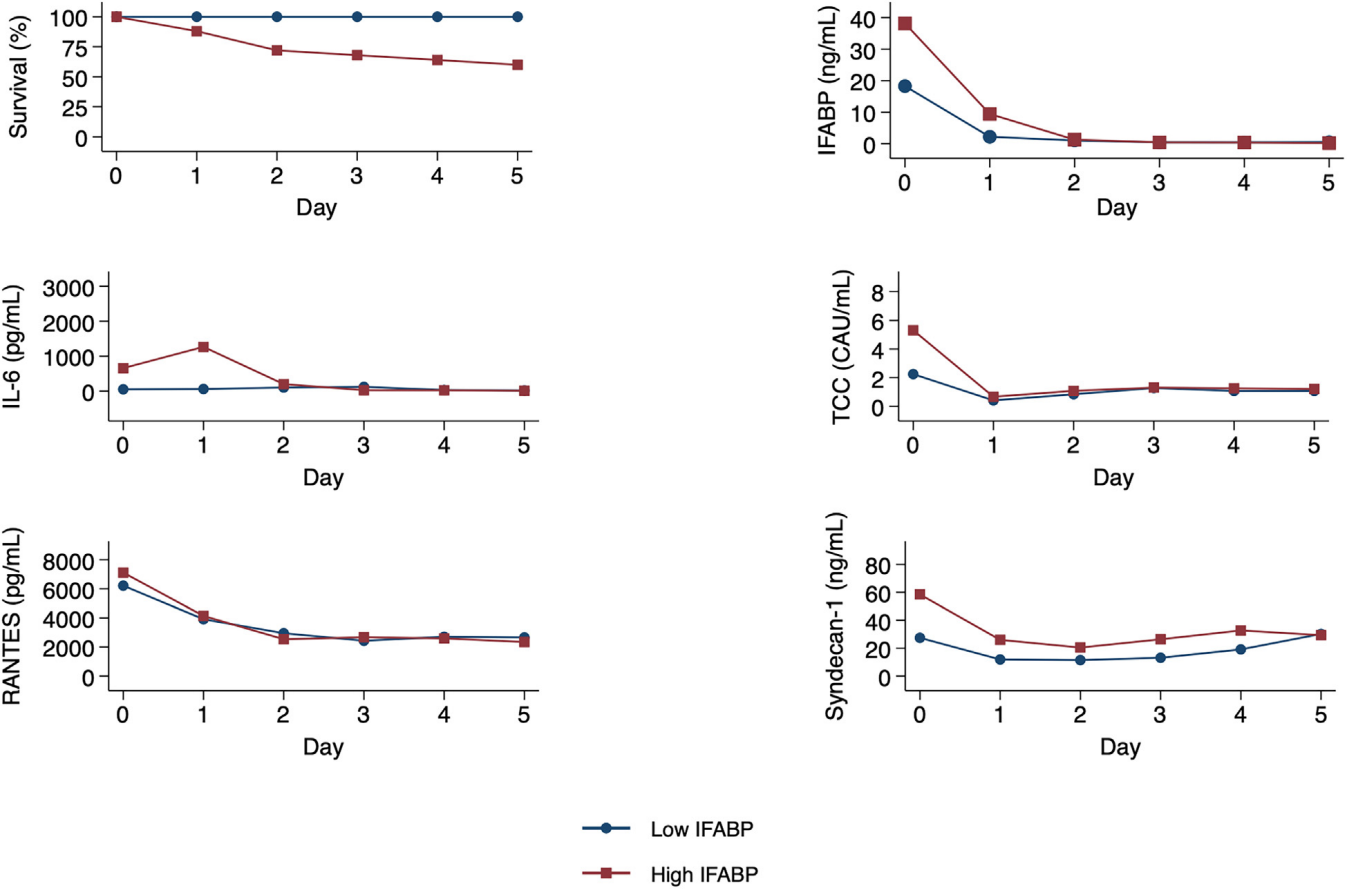
Survival and trajectories of selected biomarkers. Biomarkers are expressed as means by “low” or “high” IFABP, at the start of the first five days. Only patients still treated in ICU are included (*n* = 50, 45, 38, 31, 27 and 25 for day 0–5, respectively). Day one started mean 11 hours after admission. OHCA: Out-of-hospital cardiac arrest; IFABP: Intestinal Fatty Acid Binding Protein; IL: interleukin; RANTES: regulated on activation normal T-cell expressed and secreted; TCC: terminal complement complex.

Sixty-five consecutive patients were assessed for eligibility; seven were excluded due to immediate withdrawal of life-support, two had septic etiology, two were not in need of ICU admission, three received VAD or ECMO and one underwent immediate surgery. Fifty patients were included in the study. Patient characteristics are shown in Table 1. Detailed demographic data have been published previously.^21^, ^26^ At start of day two (mean 36 hours after admission), seven patients had died, all in the “high” IFABP group (Fig. 2), and six patients had been transferred to ward, all in the “low” IFABP group.

**Table 1:** Characteristics of successfully resuscitated OHCA patients.

	Low IFABP (*n* = 25)	High IFABP (*n* = 25)
Demographic variables		
Age	67 [54–73]	65 [52–76]
Sex, male	22 (88%)	18 (72%)
Prehospital Utstein variables		
Witnessed cardiac arrest	22 (88%)	20 (80%)
Bystander CPR	23 (100%)	21 (84%)
Shockable initial rhythm	24 (96%)	15 (60%)
Adrenaline, total dose in mg	0 [0–0]	2 [1–3]
Time to ROSC (min)	18 [10–28]	28 [20.5–35.5]
Presumed cardiac etiology	24 (96%)	18 (72%)
At admission		
Initial arterial lactate (mmol/L)	3.8 [2.1–6.6]	9.3 [5.2–12]
Certain pulmonary aspiration	2 (8%)	7 (28%)
Mechanically ventilation	18 (72%)	24 (96%)
Circulatory shock in ER*	4 (16%)	13 (52%)
Dose of noradrenaline (microg/kg/min)	0.02 [0–0.1]	0.1 [0.04–0.13]
During ICU stay		
Treatment with antibiotics	15 (60%)	15 (60%)
Tratment with inotropic drugs†	1 (4%)	9 (36%)
Ventilator, hours	49 [1–173]	65 [21–137]
Cause of death, by day 30		
Circulatory	0 (0%)	2 (8%)
Multiple organ failure	0 (0%)	3 (12%)
Irreversible brain injury	1 (4%)	9 (36%)
Other/unknown	1 (4%)	0 (0%)

The cohort is divided by the median IFABP (31.4 ng/mL). Variables are expressed as median (interquartile range [Q1–Q3]) or numbers (%). OHCA: Out-of-hospital cardiac arrest; IFABP: Intestinal Fatty Acid Binding Protein; CPR: Cardiopulmonary resuscitation; ROSC: Return of spontaneous circulation; ER: Emergency room.

* Shock defined as systolic blood pressure < 90 mmHg *or* in need of fluids and/or vasopressors to maintain systolic blood pressure > 90 mmHg. Have been published previously.

† Inotropic drugs: Adrenaline, dobutamine, dopamine.

#### Association between IFABP and inflammatory biomarkers at admission

Median of IFABP was 31.4 (IQR, 21–38) ng/mL at admission, rapidly declining thereafter (Fig. 2).^21^ The high IFABP group (i.e. >31.4 ng/mL) had higher absolute levels of cytokines (IL-1ra, IL-6, IL-8) and complement activation (TCC, C3bc) (Table 2). Differences in markers of platelet degranulation and endothelial injury were not statistically significant. The trajectories of IL-6, TCC, syndecan-1 and RANTES during the first five days are shown in Fig. 2, while C3bc, IL-1ra, IL-8, VE-cadherin and vWF are shown in Supplementary Fig. 1.

**Table 2:** Biomarkers on admission of successfully resuscitated OHCA patients.

	Low IFABP (*n* = 25)	High IFABP (*n* = 25)	*p* value
Cytokines			
IL-6 (pg/mL)	26.9 [9.6–74.0]	100.7 [23.8–447.3]	0.004
IL-8 (pg/mL)	12.1 [7.5–17.3]	58.3 [16.2–93.3]	<0.001
IL-1ra (pg/mL)	1213.5 [377–3961]	3533 [1500–9862]	0.02
Complement activation			
TCC (CAU/mL)	0.7 [0.4–2.2]	2.7 [1.0–4.7]	0.004
C3bc (CAU/mL)	10.0 [6.4–16.0]	21.0 [13.0–57.0]	0.002
Platelet degranulation			
RANTES (pg/mL)	5926 [5026–6698]	7040 [5487–8289]	0.10
Endothelial injury			
Syndecan-1 (ng/mL)	21.6 [9.9–34.7]	30.8 [15.1–79.7]	0.05
VE-Cadherin (ng/mL)	4055 (999)	3450 (1334)	0.08
vWF (U/mL)	0.79 [0.49–1.747]	1.14 [0.66–1.88]	0.26

The cohort is divided by the median IFABP (31.4 ng/mL). Variables are expressed as mean (standard deviation) and compared by Student's t-Test, or median (interquartile range [Q1–Q3]) and compared by Wilcoxon rank-sum test. OHCA: Out-of-hospital cardiac arrest; IFABP: Intestinal Fatty Acid Binding Protein; IL: interleukin; RANTES: regulated on activation normal T-cell expressed and secreted; VE: vascular endothelial; U: Unit; vWF: von Willebrand factor; TCC: terminal complement complex; C3bc: activated complement C3.

### Mediation analysis

#### Effects of IFABP on organ dysfunction at start of day two – Model 1

For SOFA-score as a dependent variable, the regression coefficient for the indirect effect through IL-6 was 0.9 (95% CI, 0.2–1.5; *p* = 0.009) SOFA-points. I.e. patients with IFABP 38 ng/mL had 0.9 points higher SOFA score than patients with 21 ng/mL. The total effect was 3.1 (95% CI, 1.6–4.6; *p* < 0.001), and the direct effect was 2.3 (95% CI, 0.7 to 3.9; *p* = 0.006). When TCC was the mediator, the indirect effect through TCC was not statistically significant (0.45 [95% CI, −0.46 to 1.36]; *p* = 0.33).

The estimated indirect effects of IFABP through IL-6 and TCC on circulatory variables were not statistically significant and/or small (Supplementary Table 2).

#### Effects of IFABP on 30-day mortality – Model 2

**Fig. 3:**
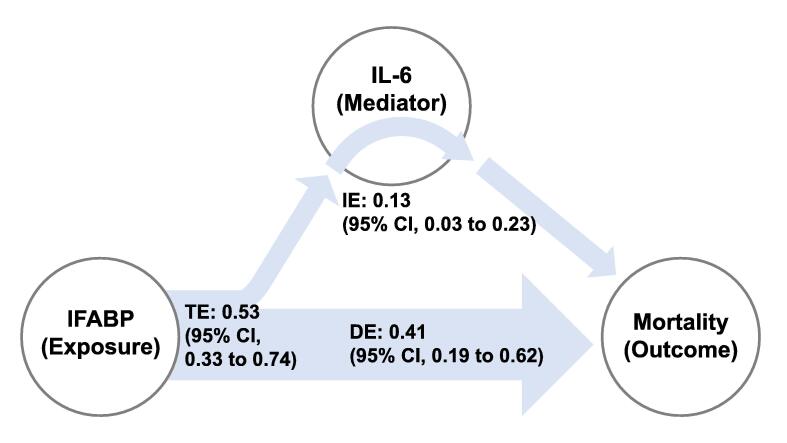
Effects of IFABP and IL-6 on 30-day mortality in causal mediation analysis (Model 2). The total effect (TE) of IFABP on mortality is the sum of the indirect effect (IE) of IFABP through IL-6 and the direct effect (DE). The reported effects are risk differences between the 75th percentile of IFABP (38 ng/mL), compared to 25th percentile (21 ng/mL). IFABP: Intestinal Fatty Acid Binding Protein; IL: Interleukin; CI: Confidence interval.

Patients on the 75th percentile of IFABP, compared to the 25th percentile, had an estimated indirect effect through IL-6 of 0.13 (RD; 95% CI, 0.03–0.23; *p* = 0.01) RD for dying within 30 days (Fig. 3). The total effect was 0.53 (RD; 95% CI, 0.33–0.74; *p* < 0.001) and the direct effect was 0.41 (RD; 95% CI, 0.19–0.62; *p* < 0.001). The effects were reduced when we added the covariates “time to ROSC” and “non-shockable rhythm” to Model 2 (Supplementary Table 3). When lactate was added as a covariate, estimates could not be computed due to non-convergence of the calculations.

When TCC was the mediator, the indirect effect was not statistically significant (0.12 [95% CI, −0.04 to 0.28]; *p* = 0.13). The total effect was 0.54 (95% CI, 0.32–0.76; *p* < 0.001) and the direct effect was 0.42 (95% CI, 0.17–0.66; *p* = 0.001).

### Sensitivity analyses

When IL-6 at day one was exchanged for IL-6 at admission, the indirect effect through IL-6 on mortality was lower and not significant. In multivariable logistic regression, using death within 30 days as the dependent variable, IFABP was associated with outcome in both models, but IL-6 and TCC in none (Supplementary Tables 4–6).

## Discussion

In this post-hoc study, IFABP was associated with cytokines and complement activation at admission, but not with other inflammatory biomarkers. Only the two biomarkers most consistently associated with mortality, IL-6 and TCC, were included in the causal mediation analyses.^20^, ^24^ Compared with the direct effects of intestinal injury on SOFA-score and mortality, the indirect effects mediated through IL-6 were significant, but substantially smaller than the direct effects. The indirect effects through IL-6 on circulatory variables, as well as all indirect effects through TCC on circulatory variables, SOFA-score and mortality, were not statistically significant and/or small.

We applied causal mediation analysis to explore if detrimental effects of intestinal injury could be mediated through inflammation. Thus, the direct effect of the exposure on outcome should not be interpreted as a causal effect, rather potential effects not attributed to the mediator. Further, we cannot firmly conclude on causal indirect effects, since the assumption of no unmeasured confounders is not likely to hold.^24^ Conversely, after analysing twenty-one inflammatory biomarkers and selecting the two biomarkers most consistently associated with outcome, few indirect effects were significant, and these were substantially smaller than the direct effects.^20^ When we added possible confounders, the indirect effects were reduced. Therefore, an important inflammatory response caused by intestinal injury seems unlikely in this cohort.

Intestinal ischemia may be a clinical concern related to low-flow and high-dose epinephrine given during resuscitation.^9^, ^32^ Intestinal ischemia is an even more pronounced concern when applying novel therapies in cardiac arrest like resuscitative endovascular balloon occlusion of the aorta (REBOA) or extracorporeal cardiopulmonary resuscitation (E-CPR).^33^, ^34^, ^35^ The presented findings and our previous paper explore the relationship between intestinal ischemia and organ failure and mortality.^21^ This relationship is not fully understood and may indicate that more underlying information is needed before we start new clinical trials. This is supported by the fact that clinical trials investigating the use of the IL-6 inhibitor tocilizumab and prehospital methylprednisolone had no effect on survival compared to placebo.^36^, ^37^

Causal mediation analysis combines regression models, and the limited mediated effects could be because of weak associations between IFABP and inflammatory biomarkers, between inflammatory biomarkers and outcome, or both.^24^ To answer this was not part of the study, but we found associations between IFABP, IL-6 and TCC. In other studies, associations between IFABP and inflammatory biomarkers have been found in malaria, but not in sepsis and cardiac surgery.^15^, ^17^, ^18^, ^19^, ^38^ Further, IFABP has been convincingly associated with mortality after cardiac arrest, while IL-6 are among the few inflammatory biomarkers which are associated with poor outcome after adjustment for confounding factors.^9^, ^39^, ^40^, ^41^, ^42^, ^43^, ^44^ Importantly, IFABP and inflammatory biomarkers could merely increase in parallel with the degree of whole-body ischaemia.^20^, ^21^

There are a few caveats with this study. First, small indirect effects through separate biomarkers could add up to a large effect in total, although IL-6 and TCC are central in the inflammatory response.^5^, ^20^ Second, perhaps only the most severely injured intestines cause a sustained inflammatory response.^45^ However, after excluding patients who died of irreversible brain injury, leaving patients with extra-cerebral organ dysfunction as assumed cause of death, inflammatory biomarkers were still not associated with mortality, while IFABP was. Third, we analysed both IFABP and inflammatory biomarkers at admission since previous studies have shown that IFABP increased before IL-6 during cardiac surgery, and both declined early.^15^, ^17^, ^18^ Both biomarkers have also declined early after cardiac arrest.^10^, ^11^, ^42^, ^43^, ^44^, ^46^, ^47^, ^48^, ^49^ In the sensitivity analysis, the effect through IL-6 at day one was less pronounced than through IL-6 at admission, suggesting against a delayed inflammatory response.

The concept of “gut origin sepsis” denotes intestinal injury leading to a “sepsis-like” syndrome in the critically ill patient.^6^ The term “sepsis-like syndrome” has also been applied on patients after cardiac arrest who share some features with septic patients, such as vasodilation, intravascular volume depletion, and endothelial injury.^5^, ^49^ We found limited effects of IFABP through inflammatory biomarkers on SOFA-score and variables reflecting vasodilation (SVR, dose of noradrenaline and MAP) and volume depletion (fluid infusion), while markers of endothelial injury were not consistently associated with outcome and excluded from the analysis.^20^ Thus, our findings indicate that intestinal injury did not lead to a “sepsis like syndrome” in this cohort, and contrast with the concept of “gut origin sepsis”.

In summary, a limited mediated effect of intestinal injury through IL-6 on organ dysfunction and death is possible. Our findings suggest, however, that intestinal injury is not a “driver” of critical illness acting through an inflammatory response after cardiac arrest.^7^, ^8^

## Limitations

The main limitations of this study are the post hoc design, single center study, and the small sample size. Specific features of this cohort, such as a high proportion of bystander CPR, could limit the generalizability. Further, the validity of IFABP has not been established in the cardiac arrest population, and although lL-6 is extensively studied, different analytical methods can make comparisons challenging. Lastly, the immune system is complex, there may be an interplay between different organ failures, and other factors not studied in this analysis may be important.

## Conclusion

To our knowledge, this is the first report on the relationship between intestinal injury and inflammation after cardiac arrest. By causal mediation analysis, effects of intestinal injury mediated through the inflammatory response on organ dysfunction and mortality were limited. Small, but significant, effects through IL-6 were noted.

## Funding

Author BHF has received PhD-funding by the Norwegian Air Ambulance Foundation.

## CRediT authorship contribution statement

**Bjørn Hoftun Farbu:** Writing – review & editing, Writing – original draft, Visualization, Project administration, Methodology, Investigation, Formal analysis, Conceptualization. **Stian Lydersen:** Writing – review & editing, Methodology, Formal analysis, Conceptualization. **Randi Marie Mohus:** Writing – review & editing, Methodology, Formal analysis. **Thor Ueland:** Writing – review & editing, Investigation, Formal analysis, Data curation. **Tom Eirik Mollnes:** Writing – review & editing, Investigation, Formal analysis, Data curation. **Pål Klepstad:** Writing – review & editing, Supervision, Methodology, Conceptualization. **Halvor Langeland:** Writing – review & editing, Methodology, Investigation, Data curation, Conceptualization.

## Declaration of competing interest

The authors declare the following financial interests/personal relationships which may be considered as potential competing interests: ‘Bjoern Hoftun Farbu reports financial support and administrative support were provided by Norwegian Air Ambulance Foundation. If there are other authors, they declare that they have no known competing financial interests or personal relationships that could have appeared to influence the work reported in this paper’.
