# Supplementary material for: The detrimental effects of intestinal injury mediated by inflammation are limited in cardiac arrest patients: A prospective cohort study
Source: Resusc Plus. 2024 Apr 17;18:100639. doi: 10.1016/j.resplu.2024.100639 (PMC11043872; doi:10.1016/j.resplu.2024.100639)
Supplement: Supplementary data 1 [file mmc1.docx]

## Supplementary appendix

### Contents

Supplementary figure 1. Trajectories of other inflammatory biomarkers.

Supplementary table 1. Commands used in Stata for mediation analyses.

Supplementary table 2. Effects of IFABP on organ dysfunction at start of day two -Model 1

Supplementary table 3. Effects of IFABP on 30-day mortality with IL-6 as mediator, including one covariate at a time -Model 2.

*Sensitivity analyses*

Supplementary table 4. Effects of IFABP on 30-day mortality with outlier in IL-6 included, and IL-6 at admission exchanged for IL-6 at day one.

Supplementary table 5. Logistic regression with death within 30 days as dependent variable, confounders included one at a time.

Supplementary table 6. Logistic regression with death within 30 days as dependent variable, hypoxic-ischaemic brain injury excluded.


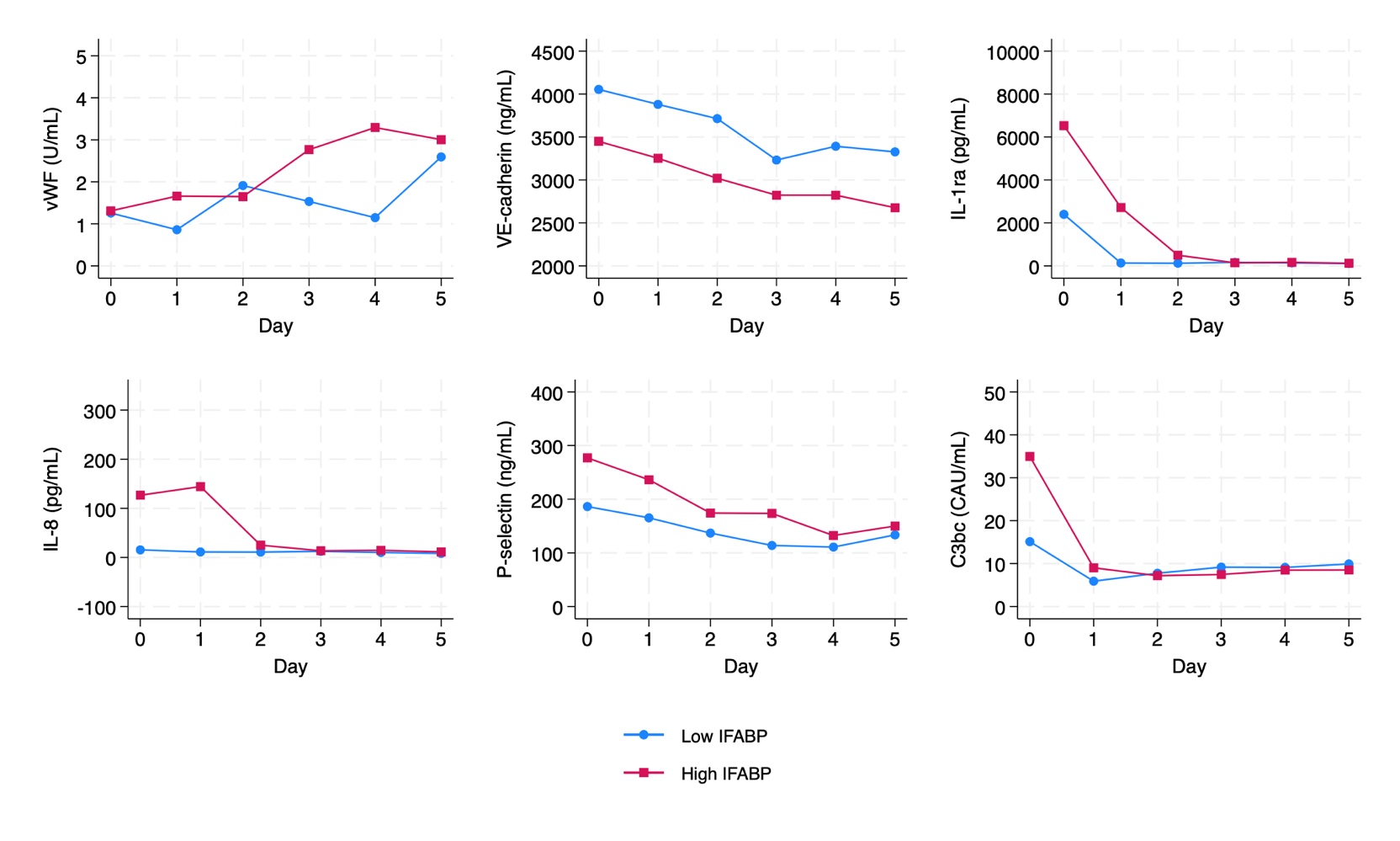


**Supplementary fig. 1 – Trajectories of other inflammatory biomarkers. Biomarkers are expressed as means by “low” or “high” IFABP, at the start of the first five days. Day zero is at admission, day one started mean 11 hours after admission. Only patients still treated in ICU are included (n=50, 45, 38, 31, 27 and 25 for day 0-5, respectively). IFABP: Intestinal Fatty Acid Binding Protein; C3bc: activated complement 3b; IL: interleukin; RANTES: regulated on activation normal T-cell expressed and secreted; TCC: terminal complement complex; VE: vascular endothelial; vWF: von Willebrand factor; U: Unit.**

| **Supplementary table 1. Commands used in Stata for mediation analyses.** | |
| --- | --- |
| *Model 1:* | mediate (“Variable of organ dysfunction”) (“IL-6” or “TCC”) (“IFABP”, continuous (21 38)) |
| *Model 2:* | mediate (“30-day mortality”, probit) (“IL-6” or “TCC”) (“IFABP”, continuous (21 38)) |
| *Model 2, with covariates* | mediate (“30-day mortality” “covariate”, probit) ([“IL-6” or “TCC”] “covariate”) (“IFABP”, continuous (21 38)) |
| All biomarkers are measured at admission, inflammatory biomarkers are log_2_ transformed. 75^th^ percentile of IFABP (38 ng/mL), compared to 25^th^ percentile (21 ng/mL).  IFABP: Intestinal Fatty Acid Binding Protein; IL: interleukin; TCC: terminal complement complex. | |

| **Supplementary table 2. Effects of IFABP on organ dysfunction at start of day two -Model 1.** | | | | | | |
| --- | --- | --- | --- | --- | --- | --- |
|  | **SOFA-score (points)** (n=44) | | **Fluids (mL/hr)** (n=36) | | | |
|  | IL-6 | TCC | IL-6 | TCC  Coeff. (95 % CI) | | |
|  | Coeff. (95 % CI) | Coeff. (95 % CI) | Coeff. (95 % CI) |  |  |  |
| Natural indirect effect | **0.9 (0.2 to 1.5)** | 0.5 (-0.5 to 1.4) | **10.8 (0.5 to 21.1)** | 5.9 (-7.1 to 18.9) | | |
| Natural direct effect | **2.3 (0.7 to 3.9)** | **2.8 (1.0 to 4.6)** | **21.2 (0.5 to 41.8)** | **33.3 (10.1 to 56.5)** | | |
| Total effect | **3.1 (1.6 to 4.6)** | **3.3 (2.0 to 4.6)** | **32.0 (10.5 to 53.5)** | **39.2 (21.1 to 57.3)** | | |
|  |  |  |  |  | | |
|  | **Cardiac output (L/min)** (n=23) | | **Noradrenaline (mikrog/kg/min)**(n=37) | | | |
|  | IL-6 | TCC | IL-6 | TCC | | |
|  | Coeff. (95 % CI) | Coeff. (95 % CI) | Coeff. (95 % CI) | Coeff.( 95 % CI) | | |
| Natural indirect effect | -0.2 (-0.6 to 0.2) | -0.5 (-1.1 to 0.0) | 0.01 (0.00 to 0.02) | 0.02 (0.00 to 0.04) | | |
| Natural direct effect | 0.4 (-0.3 to 1.1) | 0.7 (-0.2 to 1.5) | 0.02 (-0.02 to 0.07) | 0.02 (-0.02 to 0.07) | | |
| Total effect | 0.2 (-0.6 to 1.0) | 0.2 (-0.6 to 0.9) | 0.03 (-0.01 to 0.08) | 0.04 (0.00 to 0.08) | | |
|  |  |  |  |  |  |  |
|  | **MAP (mmHg)** (n=36) | | **SVR (dynes/sec/cm5) (**n=23) | | | |
|  | IL-6 | TCC | IL-6 | TCC | | |
|  | Coeff. (95 % CI) | Coeff. (95 % CI) | Coeff. (95 % CI) | Coeff. (95 % CI) | | |
| Natural indirect effect | -1.8 (-3.9 to 0.2) | **-3.7 (-5.7 to -1.7)** | 6.1 (-56.3 to 68.5) | 2.0 (-82.5 to 86.4) | | |
| Natural direct effect | 4.1 (0.0 to 8.1) | **5.7 (1.7 to 9.6)** | -15.6 (-184.2 to 153.0) | -1.7(-193.0 to 190.0) | | |
| Total effect | 2.3 ( -1.8 to 6.3) | 2.0 (-1.9 to 5.8) | -9.4 (-154.6 to 135.7) | 0.32 (-144.6 to 145.3) | | |
|  |  |  |  |  |  |  |
| Difference in regression coefficient between 75^th^ percentile of IFABP (38 ng/mL), compared to 25^th^ percentile (21 ng/mL). Statistically significant effects are presented in bold types. All biomarkers are measured at admission, inflammatory biomarkers are log2 transformed. CI: Confidence interval; Coeff.: coefficient; IFABP: Intestinal Fatty Acid Binding Protein; IL: interleukin; TCC: terminal complement complex. SOFA: Sequential Organ Failure Assessment. | | | | | | |

| **Supplementary table 3. Effects of IFABP on 30-day mortality with IL-6 as mediator, including one covariate at a time -Model 2** | | | | |
| --- | --- | --- | --- | --- |
|  |  | |  | |
|  | Risk difference  (95 % CI) | p-value |  |  |
| *Model A: “Non-shockable rhythm” included as a covariate (n=49)* |  | |  |  |
| Natural Indirect effect | 0.08 (-0.01 to 0.16) | 0.07 |  |  |
| Natural Direct effect | 0.32 (0.11 to 0.53) | 0.003 |  |  |
| Total effect | 0.40 (0.17 to 0.63) | 0.001 |  |  |
|  |  |  |  |  |
| *Model B: “Time to ROSC” included as a covariate (n=48)* |  |  |  |  |
| Natural Indirect effect | 0.09 (-0.01 to 0.19) | 0.09 |  |  |
| Natural Direct effect | 0.41 (0.18 to 0.64) | <0.001 |  |  |
| Total effect | 0.50 (0.27 to 0.73) | <0.001 |  |  |
|  |  |  |  |  |
| Risk difference between 75^th^ percentile of IFABP (38 ng/mL), compared to 25^th^ percentile (21 ng/mL). The covariates, initial non-shockable rhythm and time to ROSC, were included in the analyses one at the time (Model A and B, respectively). When we included initial arterial lactate, the estimation did not converge. All biomarkers are measured at admission, inflammatory biomarkers are log2 transformed. IFABP: Intestinal Fatty Acid Binding Protein; IL: interleukin; CI: Confidence interval; ROSC: Return of spontaneous circulation; SOFA: Sequential Organ Failure Assessment. | | | | |

### Sensitivity analyses

#### Mediation analyses: IL-6

| **Supplementary table 4. Effects of IFABP on 30-day mortality with outlier in IL-6 included and IL-6 at admission exchanged for IL-6 at day one.** | | |
| --- | --- | --- |
|  | Risk difference  (95 % CI) | p-value |
| *Model C: Outlier in IL-6 included (n=50)* |  |  |
| Natural Indirect effect | 0.16 (0.03 to 0.30) | 0.02 |
| Natural Direct effect | 0.38 (0.16 to 0.59) | 0.01 |
| Total effect | 0.54 (0.34 to 0.73) | <0.001 |
|  |  |  |
| *Model D: IL-6 day one*  *(n=45)* |  |  |
| Natural Indirect effect | 0.06 (-0.03 to 0.15) | 0.2 |
| Natural Direct effect | 0.39 (0.18 to 0.61) | <0.001 |
| Total effect | 0.45 (0.23 to 0.67) | <0.001 |
| Risk difference between 75^th^ percentile of IFABP (38 ng/mL), compared to 25^th^ percentile (21 ng/mL). Outlier in IL-6 included (Model C) and IL-6 at admission exchanged for IL-6 at day one (Model D). All other biomarkers are measured at admission. Inflammatory biomarkers are log2 transformed. CI: Confidence interval, IFABP: Intestinal Fatty Acid Binding Protein; IL: interleukin; TCC: terminal complement complex. | | |

#### Logistic regression

| **Supplementary table 5. Logistic regression with death within 30 days as dependent variable, confounders included one at a time.** | | | | | | |  |
| --- | --- | --- | --- | --- | --- | --- | --- |
|  | Univariable analysis | | | Multivariable analysis | | |  |
| **Variables** | **Unadjusted OR** | **(95% CI)** | **Pseudo R2** | **Adjusted OR** | **95% CI**  (Pseudo R2) | **p - value** |  |
| *Model E (without confounders)* |  |  |  |  | (R2= 0.42) |  |  |
| IFABP, per SD | 12.23 | (2.46 to 60.76) | 0.34 | 9.03 | (1.60 to 50.88) | 0.01 |  |
| IL-6 | 1.67 | (1.21 to 2.33) | 0.22 | 1.40 | (0.96 to 2.05) | 0.08 |  |
| TCC | 1.88 | (1.22 to 2.89) | 0.17 | 1.16 | (0.64 to 2.12) | 0.62 |  |
|  |  |  |  |  |  |  |  |
| *Model F* |  |  |  |  | (R2=0.53) |  |  |
| IFABP, per SD |  |  |  | 6.75 | (1.12 to 40.85) | 0.04 |  |
| IL-6 |  |  |  | 1.28 | (0.77 to 2.10 | 0.34 |  |
| TCC |  |  |  | 1.25 | (0.63 to 2.49) | 0.53 |  |
| **Non-shockable initial rhythm** | 20.57 | (3.62 to 116.83) | 0.25 | 16.03 | (1.43 to 180.03) | 0.03 |  |
|  |  |  |  |  |  |  |  |
| *Model G* |  |  |  |  | (R2=0.61) |  |  |
| IFABP, per SD |  |  |  | 19.04 | (1.41 to 257.05) | 0.03 |  |
| IL-6 |  |  |  | 1.09 | (0.69 to 1.76) | 0.70 |  |
| TCC |  |  |  | 1.34 | (0.66 to 2.75) | 0.41 |  |
| **Initial lactate, per SD** | 7.12 | (2.43 to 20.84) | 0.41 | 9.18 | (1.72 to 49.08) | 0.01 |  |
|  |  |  |  |  |  |  |  |
| *Model H* |  |  |  |  | (R2=0.42) |  |  |
| IFABP, per SD |  |  |  | 9.61 | (1.51 to 61.09) | 0.02 |  |
| IL-6 |  |  |  | 1.49 | (0.98 to 2.25) | 0.06 |  |
| TCC |  |  |  | 1.05 | (0.55 to 1.99) | 0.89 |  |
| **Time to ROSC, per SD** | 2.50 | (1.18 to 5.27) | 0.13 | 0.82 | (0.28 to 2.43) | 0.72 |  |
|  |  |  |  |  |  |  |  |
| All biomarkers are measured at admission, inflammatory biomarkers are log2 transformed. Confounders are included one at a time (Model E-H). OR: Odds ratio, CI: Confidence interval, SD: Standard Deviation, IFABP: Intestinal fatty acid binding protein. IL: interleukin; TCC: terminal complement complex; ROSC: Return of spontaneous circulation. | | | | | | |  |

| **Supplementary Table 6. Logistic regression with death within 30 days as dependent variable, hypoxic-ischaemic brain injury excluded.** | | | | | | |
| --- | --- | --- | --- | --- | --- | --- |
|  | Univariable analysis | | | Multivariable analysis (n=39)  Pseudo R2 =0.49 | | |
| **Variables** | **Unadjusted OR** | **(95% CI)** | **Pseudo R2** | **OR** | **(95% CI)** | **p - value** |
|  |  |  |  |  |  |  |
| IFABP, per SD | 28.22 | (1.92 to 414.59) | 0.42 | 22.04 | (1.14 to 425.25) | 0.04 |
| IL-6 | 1.73 | (1.09 to 2.76) | 0.21 | 1.15 | (0.60 to 2.20) | 0.67 |
| TCC | 2.23 | (1.19 to 4.16) | 0.26 | 1.58 | (0.63 to 3.94) | 0.33 |
| All biomarkers were measured at admission, inflammatory biomarkers were log2 transformed. OR: Odds ratio, CI: Confidence interval, SD: Standard Deviation, IFABP: Intestinal fatty acid binding protein. IFABP: Intestinal Fatty Acid Binding Protein. IL: interleukin; TCC: terminal complement complex. | | | | | | |
